# P31 An audit investigating community acquired pneumonia (CAP) antibiotic duration at Manchester University NHS Foundation Trust (MFT)

**DOI:** 10.1093/jacamr/dlaf118.038

**Published:** 2025-07-14

**Authors:** Anna Tilley, Amelia Darling, Emilia McHale, Kathryn Ashton

**Affiliations:** Manchester University NHS Foundation Trust (MFT), Manchester, UK; Manchester University NHS Foundation Trust (MFT), Manchester, UK; Manchester University NHS Foundation Trust (MFT), Manchester, UK; Manchester University NHS Foundation Trust (MFT), Manchester, UK

## Abstract

**Background:**

Community acquired pneumonia (CAP) is a subtype of acute respiratory infections acquired outside of a hospital setting.^1^ Given the prevalence of CAP infections in hospital, optimizing antimicrobial use for this indication presents a huge opportunity for improved antimicrobial stewardship. National and local antimicrobial guidelines recommend an antibiotic duration of 5 days.^2^ Shorter courses are proven to be equally as effective as longer courses, with outcomes measured including clinical stability, re-admission and mortality rates.^3,4^

**Objectives:**

To explore whether current guidelines for CAP prescribing, in relation to duration, are adhered to within MFT; and to explore rationale where there was non-concordance to identify potential improvement initiatives.

**Methods:**

Data were extracted from Epic, the Trust’s electronic patient database across two hospital sites (Manchester Royal Infirmary and North Manchester General Hospital) to an Excel spreadsheet. Antibiotic prescriptions with an indication stating CAP were identified across a 6 month period (1 June 2024–30 November 2024). Antibiotics were categorized by penicillin and non-penicillin groups. Courses prescribed for alternative respiratory infections (i.e. IECOPD, bronchiectasis) and courses that involved escalation to piperacillin/tazobactam or meropenem were excluded. The duration of each treatment course was calculated and if the patient received >5 days, reason for an extended duration was obtained using the medical notes.

**Results:**

In total, 165 antibiotic courses were included for audit. Adherence to guidelines of 5 days of antibiotics for CAP was 58% (96/165). Of the 69 (42%) courses >5 days, the reasons documented were: extra doses prescribed on discharge (*n*=35), nil reason documented (*n*=17), documented intent of 7 day course (*n*=7), symptoms not improved (*n*=7), infection markers not normalized (*n*=3). Of the 75 courses of antibiotics that were completed after discharge, 53% (40/75) adhered to 5 day guidance.

**Conclusions:**

This data shows that MFT is not adhering to the recommended guidance of 5 days of antibiotics in up to 42% of patients with CAP. The leading cause identified was extra doses prescribed at discharge, indicating that inpatient and discharge durations are not cumulatively added appropriately. Additionally, while extended antibiotic courses may sometimes be justified, our results show that the documentation in medical notes was lacking. It is important to acknowledge the limitations of retrospective data collection, as it does not allow direct interaction with clinical teams. Other limitations were that patient comorbidities and outcomes weren’t assessed. Ongoing education on the effectiveness of shorter antibiotic courses is crucial to support antimicrobial stewardship, to reduce unnecessary or prolonged exposure to antibiotics. Additionally, promoting documentation in medical notes and ensuring pharmacists play an active role in reviewing and challenging discharge prescriptions are key to addressing prescribing barriers. Consideration is also being given to implementing an order set, a digital tool aimed at standardizing antimicrobial prescribing in alignment with current guidelines.
